# Stereotype Awareness, Self-Esteem and Psychopathology in People with Psychosis

**DOI:** 10.1371/journal.pone.0088586

**Published:** 2014-02-11

**Authors:** Catherine van Zelst, Martine van Nierop, Margreet Oorschot, Inez Myin-Germeys, Jim van Os, Philippe Delespaul, for GROUP

**Affiliations:** 1 Maastricht University Medical Centre, South Limburg Mental Health Research and Teaching Network, EURON, Maastricht, The Netherlands; 2 Yulius Academy, Yulius, Organisation for Mental Health, Dordrecht, The Netherlands; 3 King's College London, King's Health Partners, Department of Psychosis Studies, Institute of Psychiatry, London, United Kingdom; 4 Mondriaan Mental Health Trust, South Limburg, Maastricht/Heerlen, The Netherlands; King's College London, United Kingdom

## Abstract

**Introduction:**

Stigma is an important environmental risk factor for a variety of outcomes in schizophrenia. In order to understand and remediate its effects, research is required to assess how stigma experiences are processed at the level of the individual. To this end, stereotype awareness (SA) with respect to people with mental illness and their families was explored in persons with psychotic disorder.

**Method:**

Data from the Dutch Genetic Risk and OUtcome of Psychosis project (GROUP) were analyzed. SA was measured using scales that assess a respondent's perception of common opinions about people with a mental illness and their families.

**Results:**

People with higher level of self-esteem were less aware of stereotypes about patients and families. People with more severe psychopathology reported more awareness of stereotypes about families, not about patients.

**Conclusion:**

Enhancing psychological resources, by increasing self-esteem and the ability to cope with symptoms, can be targeted to diminish stereotype threat and improve stigma resilience. Interventions can be tailored to individual differences to increase their impact. Furthermore, in order to diminish detrimental consequences of negative stereotypes, mental health professionals, health educators and experts by experience can inform the public about mental illness and stigma.

## Introduction

Mental illness is surrounded by negative stereotypes. Schneider defined stereotypes as “qualities perceived to be associated with particular groups or categories of people” [1]. When stereotypes are related to group characteristics, they can lead to public stigma; self-stigma may arise when they relate to beliefs about the self [2]. Mental illness related stereotypes center around being (un)responsible, dangerous and dependent, having a poor prognosis and poor social skills [3] and, at the positive side, being creative [4]. According to Ajtony [5], stereotyping is not inherently wrong; it is a natural function of the human/cultural mind and is therefore morally neutral in itself. However, uncritical stereotyping can result in negative consequences for individuals and society. This plays a central role in the development, justification, maintenance, and perpetuation of stigmatization [6], and contributes to discrimination.

The ‘modified labeling theory’ by Link and colleagues [7] considered societal conceptions of devaluation-discrimination of “mental patients” as the first step in the labeling process. People labeled with psychiatric problems who live in a culture where stereotypes about mental illness prevail may anticipate and internalize attitudes that reflect devaluation and discrimination [8]. Once labeled, an individual's societal conceptions become relevant to the self, altering the individual's response (cognitive, emotional and behavioral) in daily life through self-stigma.

Corrigan, Watson & Barr [9] defined Link's process of perceived discrimination as *stereotype awareness* (SA): “the person is aware of the general negative beliefs about mental illness held by one's culture” (p.876). This concept can be assessed with the Devaluation of Consumers Scale and the Devaluation of Consumers Families Scale (DCS and DCFS) [10].

### Stigma vulnerability and stereotype awareness

Anyone - with or without mental health complaints - is biased to a degree in how feedback is perceived and interpreted. The beliefs one holds about stereotypes in society, or “what most people think”, may be biased. The extent to which stereotypes are prevalent in society can be over- or underestimated easily. Negative schemas and psychopathology may increase this bias. Overestimation of the presence of stereotypes in society or attaching more value to them, or to the belief of “what most people think” may indicate stigma vulnerability. Psychological and social skills that protect against negative schemas, negative underlying assumptions or bias in information processing and perception of stereotypes (or SA) may help an individual to become more stigma-resilient. Rüsch and colleagues [11] state that identifying vulnerability and resilience factors to stress due to stigma can help reduce the impact of stigma on persons with schizophrenia and other diagnoses of mental illness. In this study, we explore the associations of self-esteem and psychopathology with the individual's awareness of stereotypes.

Previous work on SA and self-esteem showed that self-esteem can be influenced negatively by SA [12]. Furthermore, cognitive processing, emotional thresholds and behavioral responses that are related to psychopathology, may further influence stigma susceptibility. Both stereotypes about patients as well as their families may be relevant to people with psychosis. Therefore, we aimed to investigate SA about patients as well as SA about their families, self-esteem and psychopathology in this group.

## Methods

Data pertain to second wave measures of the Genetic Risk and OUtcome of Psychosis (GROUP) study, an ongoing longitudinal multicenter study in Europe. In selected representative geographical areas of the Netherlands and (Dutch speaking part of) Belgium, patients were identified through clinicians working in regional psychosis departments or academic centers. The patients presenting consecutively at these services either as outpatients or inpatients were recruited for the study.

### Ethics statement

Persons identified as potentially eligible and deemed capable of providing informed consent by their clinician were given detailed explanation of the study procedures and were asked for written informed consent for detailed assessment and for contacting their first-degree family members (brothers, sisters, parents). Written informed consent was also obtained from the next of kin, caretakers, or guardians of those aged 16–17 years. Before written informed consent was obtained, persons had the opportunity to think about and ask questions about participation. They could talk about the study with an independent physician who was not involved in the study. All potential participants who declined to participate or otherwise did not participate were eligible for treatment (if applicable) and were not disadvantaged in any way in the case of non-participation. The study protocol was approved by the Ethical Review Board of the University Medical Centre Utrecht.

### Sample

For the current analyses, we used the subsample of the second wave of the GROUP Maastricht patients sample (N = 219). This subsample has add-on scales measuring self-esteem and SA. Data of patients with at least 70% answers on SA-scales were used. Inclusion criteria for the Maastricht add-on study were: age range of 16–50 years (at first wave), clinical diagnosis of psychotic disorder according to the Diagnostic and Statistical Manual of Mental Disorders, fourth edition (DSM-IV) criteria [13], good command of the Dutch language and able and willing to give written informed consent.

### Materials

*Stereotype awareness* was measured using the Devaluation of Consumers Scale (DCS, 8 items) and the Devaluation of Consumers Families Scale (DCFS, 7 items) [10]. These scales assess a respondent's perception of what most other people believe about people with a mental illness (DCS) and their families (DCFS). We use two scales because they assess slightly different domains. All items are rated on a 4-point Likert Scale: strongly disagree ( = 1), disagree, agree, strongly agree ( = 4). In the statistical analyses, mean scores of the scales were used as outcomes (overall scores). The higher the score on the DCS or DCFS, the more the person is aware of the general negative beliefs about mental illness held by one's culture.

*Self-esteem* was measured using the 10-item Rosenberg Self-Esteem scale (RSES) [14]. Items are rated on a 4-point Likert Scale: strongly disagree ( = 0), disagree, agree, strongly agree ( = 3). We used the individual's mean item score for analyses. A higher mean score indicates higher self-esteem.

*Psychopathology* was measured with an extended version of the Brief Psychiatric Rating Scale (28-item BPRS) [15]–[17]. Information on both frequency and severity of symptoms in the two weeks before the interview was used for scoring. Items are rated on a 7-point ordinal scale. Analyses used the individual's mean score of all 28 items.

### Statistical analysis

Linear regression analysis was conducted with scores on DCS and DCFS respectively for the assessment of stereotype awareness as dependent variables and as independent variables, the RSES as a measure of self-esteem (in model 1), to which was added BPRS severity in order to assess associations with psychopathology (model 2). Gender, age, illness duration and ethnicity were added as *a priori* confounders in all analyses. Statistical analysis was carried out using STATA 11.2 [18].

## Results

186 patients filled in more than 70% of items in the DCS, and 184 did so for the DCFS. Of the 186 patients, 129 were men (69%). Mean age was 29.8 years (range 18–53). Most patients were diagnosed with psychosis spectrum disorder (98%). Mean duration of illness was 7.7 years (range 2.1–29.1). 89% was of white ethnic group, 8% of mixed ethnic group, 1% of Moroccan ethnicity, 1% of Surinamese ethnicity, and 1% of other ethnic group. BPRS overall score was 1.5 (range 1–3.2), RSES overall score was 2.0 (0.5–3.0). On the devaluation scales, mean overall scores were 2.6 (1–4) for DCS and 2.2 (1–3.7) for DCFS. The correlation between DCS and DCFS overall scores was 0.56. The correlation between RSES score and BPRS score was 0.36.

### Stereotype awareness

Cronbach's alphas of the DCS and DCFS were respectively 0.83 and 0.86, indicating good reliability. A principal component analysis (PCA) with oblique rotation (oblimin) was conducted to explore associations in the item set. We identified factors with eigenvalues larger or equivalent to 0.9. This analysis revealed one factor for DCS and two factors for DCFS. Eigenvalues of these factors were 3.84 for DCS and 3.81 and 0.94 for DCFS.

Studying the extent to which patients agreed and disagreed with statements, the mean percentage of agreement with the DCS-items was 58%, ranging from 39% for the item “Most people feel that having a mental illness is worse than being addicted to drugs” to 71% for the item “Most people think less of a person who has been a patient in a mental hospital” (Table 1). On the DCFS, most answers indicated *disagreement* with the statements. The mean percentage of *agreement* was 31% ranging from 26% for both of the items “Most people in my community would rather not be friends with families that have a relative who is mentally ill living with them” and “Most people do blame parents for the mental illness of their children” to 41% for the item “Most people would not treat families with a member who is mentally ill in the same way they treat other families” (Table 2).

**Table 1:** Patient's responses on statements of the DCS (N = 186).

DCS		Disagree or Strongly disagree (%)	Agree or Strongly agree (%)
1	Most people would not accept a person who once had a serious mental illness as a close friend	58.82	41.18
2	Most people think that a person with a serious mental illness is dangerous and unpredictable	41.18	58.82
3	Most people feel that having a mental illness is worse than being addicted to drugs	60.75	39.25
4	Most people look down on someone who once was a patient in a mental hospital	33.33	66.67
5	Most employers will not hire a person who once had a serious mental illness if he or she is qualified for the job	33.87	66.13
6	Most people think less of a person who has been a patient in a mental hospital	29.03	70.97
7	Most people feel that entering psychiatric treatment is a sign of personal failure	45.16	54.84
8	Most young women would not marry a man who has been treated for a serious mental disorder	37.63	62.37
	Mean percentage of participants	42.5	57.5

**Table 2:** Patient's responses on statements of the DCFS (N = 184).

DCFS		Disagree or Strongly disagree (%)	Agree or Strongly agree (%)
9	Most people in my community would rather not be friends with families that have a relative who is mentally ill living with them	74.46	25.54
10	Most people believe that parents of children with a mental illness are not as responsible and caring as other parents	68.48	31.52
11	Most people look down on families that have a member who is mentally ill living with them	69.02	30.98
12	Most people believe their friends would not visit them as often if a member of their family were hospitalized for a serious mental illness	68.48	31.52
13	Most people would not treat families with a member who is mentally ill in the same way they treat other families	59.24	40.76
14	Most people do blame parents for the mental illness of their children	74.46	25.54
15	Most people would rather not visit families that have a member who is mentally ill	71.20	28.80
	Mean percentage of participants	69.3	30.7

### Self-esteem

The Cronbach's alpha of the RSES was 0.89 indicating good reliability. Almost all participants (98%) agreed with the statement “I feel that I have a number of good qualities”. On the other hand, almost half of participants expressed (strong) agreement on the items “I wish I could have more respect for myself” and “I certainly feel useless at times”.

### Linear regression analysis

Results of linear regression analysis showed that in all models, self-esteem was significantly associated with SA (beta  =  −0.37, p7<0.001 in model 2 for DCS and beta  =  −0.32 in model 2 DCFS). More self-esteem was associated with less SA. Age was significantly associated with DCS-score (beta  =  0.17, p<0.001 in model 2 DCS). Psychopathology was significantly associated with DCFS-score (beta  =  0.21, p = 0.009 in model 2), but not with DCS-score. In both models of DCS and DCFS there were no significant associations between SA and 1) gender, 2) illness duration, or 3) ethnicity. The explained variance, R?, ranged from 0.18 (model 1 DCFS) to 0.22 (model 2 DCS), indicating that 18 to 22% of variance in SA was explained by the independent variables (Table 3).

**Table 3:** Results of linear regression analyses on stereotype awareness (SA).

	DCS			DCFS		
	ß	p		ß	p	
**Model 1**	R^2^ = 0.21			R^2^ = 0.18		
Self-esteem (RSES)	−0.41		<0.001*	−0.39		<0.001*
Gender	0.05		0.481	0.05		0.483
Age	0.16		0.039*	0.13		0.103
Illness duration	−0.03		0.701	−0.15		0.064
Ethnicity	−0.01		0.934	−0.03		0.660
**Model 2**	R^2^ = 0.22			R^2^ = 0.21		
Self-esteem (RSES)	−0.37		<0.001*	−0.32		<0.001*
Psychopathology (BPRS)	0.12		0.133	0.21		0.009*
Gender	0.07		0.324	0.11		0.145
Age	0.17		0.029*	0.13		0.086
Illness duration	−0.03		0.738	−0.13		0.088
Ethnicity	−0.02		0.763	−0.06		0.413

## Discussion

This is, to our knowledge, the first study that shows an association between stereotype awareness about patients (DCS) and their families (DCFS) and self-esteem in people with psychotic disorder. Results show that decreased self-esteem is associated with increased awareness of stereotypes of patients and their family members. Furthermore, (severity of) psychopathology is associated with stereotypes about family members of patients with mental illness. This reflects that not only patients but also their families can be the subject of stereotyping and stigmatization, while the awareness of stereotypes concerning families is associated with psychopathology. A non-significant association between SA and severity of psychopathology in the same direction was found in SA concerning patients.

Being aware of stereotypes held by the public (“most people” in society) with respect to patients' families, may also be a source of distress. It may be an indicator of courtesy stigma [19]; the process of being stigmatized by virtue of association with a stigmatized individual [20]. When people with mental illness not only perceive stereotypes about patients but also about families, stigma experiences may be even more pervasive for the individual. However, in the present study, patients disagreed (disagree/strongly disagree) with most of the statements on the DCFS, indicating that they did not perceive stereotypes about families of people with mental illness to be held by “most people” in society. Most participants perceived less stereotypes about family members of people with mental illness, than stereotypes about patients.

Patients agreed the most with item 4 (“Most people look down on someone who once was a patients in a mental hospital”) and 6 (“Most people think less of a person who has been a patient in a mental hospital”) of the DCS. According to Struening and colleagues [10] these items indicate a judgmental reaction to consumers who have experienced hospitalization for mental illness, ascribing them a lower status position. These responses are considered put-downs or expressions of rejection or criticism [10].

The study showed that higher self-esteem was associated with less SA. Positive self-esteem is not only seen as a basic feature of mental health, but also as a protective factor that contributes to better mental health and positive social behavior through its role as a buffer against the impact of negative cognitions, emotions and dysfunctional behavioral responses. Self-esteem represents a motivational force that influences perceptions and coping. In the context of negative messages and stressors, positive self-esteem can have various protective functions [21]. Protective factors interact with risk to modify its effects in a positive direction [22].

In a model by Watson and coworkers [23], SA is viewed as one of the components in the process of self-stigma. Other components are stereotype agreement - endorsing the same stereotypes perceived to be common in the public - and self-concurrence - when people believe that culturally internalized beliefs in fact apply to them [9]. In the present study, high self-esteem is viewed as a protective factor leading to resilience, while low self-esteem may be a vulnerability factor for the experience of stigma. Of course, no causal attributions are possible given the cross-sectional nature of the data.

Brohan and colleagues [24] found that 42% of people with schizophrenia or other psychotic disorders experienced self-stigma in moderate to high levels. Dealing with SA may be a useful way to diminish self-stigma experiences of the individual. While SA can be viewed as an indicator of public stigma, people may over- or underestimate the stereotypes in the public.

A sense of self-worth and a belief that one can control one's destiny and life events, actual power, and righteous anger and community activism are important elements of empowerment [25]. Rogers and colleagues [25] indicate that programs aimed at promoting empowerment must focus on, for example, increasing self-esteem and self-efficacy, decreasing feelings of powerlessness, and increasing feelings of power. Stigma resilience and empowerment may be promoted in therapeutic and/or educational programs, in which experts by experience can play an important role. However, functional strategies to cope with stigma have to be applied in stigmatizing situations in everyday life. The social network of the individual is important in this respect. A limited social network may contribute to the vulnerability to internalize stigmatizing attitudes and to more strongly perceive devaluation and discrimination [26]. Furthermore, individual as well as situational characteristics are important in the experience of stigma: The response to stigma, of an individual with mental illness, may consist of diminished self-esteem and self-efficacy, righteous anger and empowerment, or relative indifference, depending on the parameters of the situation [27]. Corrigan and Kleinlein [28] state that persons with intact self-esteem will respond to stigma with indifference or indignation depending on their identification with the generic group of people with mental illness. Peterson and Barnes [29] indicate: “If people with experience of mental illness are encouraged to empower themselves, their self-efficacy and self-esteem will increase, thus combating self-stigma”.

### Limitations

Data were derived from a cross-sectional survey. Therefore, self-esteem, psychopathology and SA were measured at the same moment, precluding conclusions about temporal relationships and causality. The results may be influenced by the fact that most participants (69%) were male and participants reported relatively low levels of psychopathology (BPRS) in the two weeks before measurement. Moreover, people were diagnosed with a psychosis spectrum disorder, which limits our findings to this (broad) group of individuals. Also, perception of the respondent of “most people's” beliefs may be influenced by, for example, alterations in social cognition such as theory of mind.

### Practical implications

Self-esteem and SA may be usefully targeted in anti-stigma interventions. This strategy may be of particular interest for people with early symptoms of psychosis. Targeted interventions at this stage may alter illness outcomes. Perceived stereotyping may exacerbate early psychopathology in the case of incipient illness, or increase the probability of relapse in case of established illness, for instance, by inducing a vicious circle of self-blame, attributing negative feedback to the self or by increasing stigma consciousness [30]. Thus, in the phase of early intervention, one of the factors that may prevent transition to more severe psychopathology is to increase resilience in subjects with a high level of awareness of stereotypes. Interventions can help people to deal with stigma, to increase self-esteem, and to become more stigma-resilient [30].

Future research can inform us on SA, about people with mental illness and their families, in people with or without other mental health problems or diagnoses. It may also further inform us on associations with for example self-esteem and other protective factors, as well as psychopathology. Research on gender and stigma-related factors [31], [32] can be elaborated to customize gender-specific interventions. Gender is a source of stereotyping in itself. Therefore, research on its direct and indirect effects on stigmatization in people with mental illness is required.

The consequences of stereotype *threat* [33] may be worse than SA, for example by increasing stress and interfering with daily life functioning. In future studies, it is important to include situational characteristics that can bring about stereotype threat in the flow of daily life, measured, for example, with the Experience Sampling Method [34]–[36].

## Conclusion

Enhancing psychological resources, focusing on self-esteem and the ability to cope with symptoms, may be relevant targets to increase stigma resilience and empowerment. A higher level of self-esteem may be a protective factor, and coping with symptoms may reduce the consequences of stigma experience. SA may precede or co-occur with self-stigma and can be targeted in conjunction with other aims of treatment. Interventions can be tailored to individual differences to increase their effect. In tandem with the individual approach in patients, the public should be informed about mental illness and stigma, including the negative consequences of uncritical stereotyping.
